# Supplementary material for: Optimization of adaptive filter control parameters for non-invasive fetal electrocardiogram extraction
Source: PLoS One. 2022 Apr 11;17(4):e0266807. doi: 10.1371/journal.pone.0266807 (PMC9000127; doi:10.1371/journal.pone.0266807)
Supplement: Attachment — Submitted filename: Response to Reviewers.pdf [file pone.0266807.s001.pdf]

Original Manuscript ID: PONE-D-21-24207

Original Article Title: **"**Optimization of Adaptive Filter Control Parameters for Non-Invasive Fetal Electrocardiogram Extraction"

To: PLoS ONE Editor

Re: Response to reviewers

Dear Editor,

Thank you for you feedback to our manuscript. We would like to take this opportunity and thank the reviewers for reviewing our manuscript. Below, we are uploading point-by-point response to the comments of all reviewers. The changes were highlighted in red.

Best regards,

Radana Kahankova et al.

Responses to Editor:

Comment 1. Please ensure that you refer to Figure 9 in your text as, if accepted, production will need this reference to link the reader to the figure.
Authors' action: We added reference to Fig. 6 (lines 279, 294) and Fig. 9 (line 417).

Comment 2. We note you have included a table to which you do not refer in the text of your manuscript. Please ensure that you refer to Tables 1, 2, 3, 4, and 5 in your text; if accepted, production will need this reference to link the reader to the Table.
Authors' action: We went through the manuscript, all tables are mentioned: Tab1 (lines 102 and 117), Tab2 (line 278), Tab 3 (lines 326, 328, 338, 345), Tab4 (line 363), Tab5 (lines 431, 434, 438, 474)

Reviewer #1

This paper is focused on the design, implementation and verification of a novel method for the optimization of the control parameters of different hybrid systems used for non-invasive fetal electrocardiogram (fECG) extraction. This paper is generally well written with sufficient analysis. It can be accepted if the following concerns can be addressed.

Comment 1: Please ensure all the variables have been defined clearly in the paper.

Authors' answer: Thank you, for your suggestion. We went through the text and corrected parts where the variables were not sufficiently described.

Authors' action: We found the cases where the variables were not sufficiently defined and corrected them.

Comment 2: The parameter tuning is critical to the performance of the discussed method, so more details should be given on how to select the parameters.

Authors' answer: Thank you, for your suggestion, this will be added.

Authors' action: We added more details regarding selection of the parameters to be tuned.

Comment 3: Least squares-based methods such as RLS and LMS have been widely used for system parameter identification, e.g., DOI: 10.1109/JESTPE.2021.3098836; DOI: 10.1109/TTE.2020.2994543. Such works can be included for brief discussion.

Authors' answer: Thank you, for your suggestion. We mentioned system parameter identification as one of the use cases of LMS algorithms. The works are from quite different area not related to biosignals, so we did not make extended discussion of them.

Authors' action: We mentioned system parameter identification as one of the use cases of LMS algorithms (line 133). We included the mentioned works as references.

Comment 4: Least squares-based identification is vulnerable to the noises on system input. Noise-compensated versions of them have been proposed in the literature for noise-immune and unbiased identification, e.g., DOI: 10.1109/TIE.2019.2962429; DOI: 10.1109/TIE.2021.3063968. Such works can be mentioned for completeness and maybe as a future work.

Authors' answer: Thank you, for your suggestion. We mentioned this in the introduction but since the mentioned works and the approach is from a completely different field that is not related to proposed study, we did not include it into the future work in further discussions.

Authors' action: We added this information and reference to the introduction.

Comment 5: The conclusions can be further improved. Primary findings with statistical results should be given.

Authors' answer: Thank you, for your suggestion. Indeed, the conclusion was not sufficient.

Authors' action: We expanded the conclusion section.

Reviewer #2

This article raises a very interesting topic and will definitely be discussed further in the future. And after applying a minor change can be published

Comment 1: More literature needs to be surveyed.

Authors' answer: Thank you, for your suggestion, we extended the literature survey for the latest research outcomes and added them to the introduction and comparison of the results.

Authors' action: The literature survey was extended.

Comment 2: Discussion is short. Please express it more detailed.

Authors' answer: Thank you, for your suggestion, the discussion was not structured right and seemed short.

Authors' action: We revised the discussion section.
