# Supplementary material for: Optimization of adaptive filter control parameters for non-invasive fetal electrocardiogram extraction
Source: PLoS One. 2022 Apr 11;17(4):e0266807. doi: 10.1371/journal.pone.0266807 (PMC9000127; doi:10.1371/journal.pone.0266807)
Supplement: Attachment — Submitted filename: Response to Reviewers2.pdf [file pone.0266807.s002.pdf]

Original Manuscript ID: PONE-D-21-24207

Original Article Title: **"**Optimization of Adaptive Filter Control Parameters for Non-Invasive Fetal Electrocardiogram Extraction"

To: PLoS ONE Editor

Re: Response to reviewers

Dear Editor,

Thank you for you feedback to our manuscript. We would like to take this opportunity and thank the reviewers for reviewing our manuscript. Below, we are uploading point-by-point response to the comments of all reviewers. The changes were highlighted in red.

Best regards,

Radana Kahankova et al.

Responses to Editor:

Comment 1. One of the reviewers has some concern about the novelties of paper; please revise the paper according the comments.
Authors' action: We revised the paper according the comments.

Reviewer #2: What is the difference between your work and the method presented in the following article?

Katerina Barnova et al. System for adaptive extraction of non-invasive fetal electrocardiogram, Applied Soft Computing, Volume 113, Part B, 2021, 107940, ISSN 1568-4946, https://doi.org/10.1016/j.asoc.2021.107940.

Authors' answer: In the proposed work, we tested and optimized in total of 5 algorithms in hybrid fECG extraction system: ADALINE, LMS, SE-LMS, RLS and FTF. The combinations of ICA-ADALINE and ICA-SELMS and have not been used in the literature before. In the paper of Barnova you mentioned, the focus is fully on the ICA-FTF-CEEMDA extraction system. However, in our results, the FTF algorithm is achieving poorest results when used alone, i.e. without final processing using CEEMDA (method who's settings also need to be optimized). In our paper, the ADALINE method is most powerful when

used alone. Therefore, it could be further improved when additional processing enhancing the fetal R peaks was used (such as CEEMDA method). Further, Barnova in her paper admits that the limitation of her work is the need for optimal parameter setting of both FTF and CEEMDA algorithms (see table 8 summary in her paper). Contrary to paper of Barnova et al., we did not focus only on one method but on finding a way to optimize them all so they function well even without additional postprocessing methods – i.e. optimizing them for fECG extraction (as stated in the title of the article).

Authors' action: We added the information regarding this paper to the manuscript and highlighted the limitations.
